# Social and medical needs of rare metabolic patients: results from a MetabERN survey

**DOI:** 10.1186/s13023-021-01948-5

**Published:** 2021-08-03

**Authors:** Sylvia Sestini, Laura Paneghetti, Christina Lampe, Gianni Betti, Simon Bond, Cinzia Maria Bellettato, Scarpa Maurizio

**Affiliations:** 1 Italian Association of Patients With Alkaptonuria (aimAKU), Siena, Italy; 2 Regional Coordinating Center for Rare Diseases, MetabERN, Udine University Hospital, Udine, Italy; 3 Center for Rare Diseases Giessen (ZSEGI), Department of Child Neurology, Justus-Liebig University, Giessen, Germany; 4 Department of Economics and Statistics, University of Siena, Siena, Italy

**Keywords:** Rare diseases, Inherited metabolic diseases, IMDs, Social services, Psychological support

## Abstract

**Background:**

Many surveys have been performed over the years to assess the medical and social requirements of patients with a rare disease, but no studies have focused specifically on patients in Europe or with an inherited metabolic disease (IMD). To obtain a comprehensive overview of the social and psychological status and needs of IMD patients, especially in Europe, the European Reference Network for Hereditary Metabolic Disorders (MetabERN) has performed a dedicated survey among its metabolic patients.

**Results:**

A total of 924 patients and caregivers responded to the questionnaire. Most participants were from 25 European countries, with Spain, Italy, and Germany being the most represented; only eight participants were extra-European. The survey showed that most social assistance services, from free educational/development services for those with intellectual disability to transition from childhood to adult care and job placement support, are available for a limited number of patients or are unknown to the majority of patients or their parents/caregivers. Similarly, psychological assistance for the patient or the parent/caregiver is available for a small fraction of respondents, despite the fact that the majority considers this type of support necessary for both the patient and the caregiver. In addition, for most IMD patients local specialised or emergency medical assistance is lacking, although national clinical pathways are defined, and medical professionals of reference are readily available when needed. Lastly, while most national health services in Europe cover all or part of the expenses for medications, medical devices, food supplements, dietary integrators, physiotherapy, and speech therapy, significant gaps in the economic support for healthcare and other expenses still exist.

**Conclusions:**

Overall, our survey reveals a widespread lack of social, psychological, and economic support for IMD patients in Europe. More needs to be done to provide daily assistance to IMD patients in order to alleviate the burden on caregivers and to allow patients to become independent and productive adults. Where support is actually available locally or nationally, most IMD patients are not aware of it, so an active dissemination of this information among the metabolic community is essential.

**Supplementary Information:**

The online version contains supplementary material available at 10.1186/s13023-021-01948-5.

## Background

Inherited Metabolic Diseases (IMDs) are rare disorder caused by defects in biochemical pathways. At present, IMDs include over 1400 genetic diseases [1], which are classified in 130 biochemical groups based on the affected metabolic pathway [2]. While the incidence of a single IMD is low (from 1:1,000,000 to 1:10,000), the global incidence of all IMDs taken together is high, going from 1:2500 to 1:800 [3–8].

The clinical signs and symptoms and the course of the disease can vary a lot among the various IMDs: from a slow progressive disorder that is evident only in adulthood, to acute and potentially lethal metabolic decompensation shortly after birth [9]. Since IMDs are chronic and progressive diseases which can involve multiple organs, early diagnosis and treatment are crucial to delay or even stop the disease progression. However, diagnosis is often delayed due to the fact that, in most cases, the initial symptoms of IMDs are unspecific and common to other more frequent diseases. This may cause irreparable damage to the patients’ organs, triggering organ failure or dysfunction.

As rare, chronic, and complex diseases, IMDs require the constant medical assistance of experienced and highly specialised multidisciplinary teams: regular visits to different specialists and/or therapies are necessary to maintain the health and quality of life of the patients, and numerous adjustments to treatment and care are required over time as the patient grows and changes through the various phases of life. In addition, IMDs often cause mental and/or physical disability. As such, the life of IMD patients and their caregivers is strongly impacted by the disease, not only in physical, but also in psychological and social terms. Indeed, besides the obvious health challenges, patients with a rare disease also experience social difficulties and exclusion, which seriously affect their dignity and autonomy: access to education, employment, leisure activities, transport, adapted housing, and bank credit is difficult and limited [10]. This generates the need for social services, particularly to support the patients’ independence; however, most rare disease patients report inadequate preparation of social service professionals and largely unmet needs in this department [11]. As a consequence, about one third of rare disease patients feel discriminated [10]. In addition, the uncertainty about the evolution of the disease, the pain or other distressing aspects of the condition, other people’s lack of understanding and misconceptions about rare diseases generate psychological distress in patients [12]. In fact, the mental health of those with a rare disease often deteriorates and is worse compared to the general population [11]. To alleviate or prevent these problems, social support, intended as the possibility to share experiences and receive emotional support, is essential [12].

The parents and/or caregivers of IMD patients are often overlooked, but they also experience difficulties and present common needs and concerns: from acquiring specific information about the disease and its care, to financial worries for the medical expenses; from feeling lonely and isolated, to sustaining the physical and emotional burden of caring for a person with a chronic rare disease [13]. In this context, social support given in the form of emotional (e.g., encouragement), informational (e.g., advice and guidance), and tangible support (e.g., financial help) is crucial to allow an optimal adjustment to the illness, reduce the associated stress, and guarantee the wellbeing of the parent/caregiver [13]. This aspect is important also from a gender perspective: many studies have shown that women are the main caregivers for rare disease patients, and experience greater levels of stress and depression, and poorer general health [11, 13]. This reflects the traditional patriarchal societal view in which men are expected to provide financial stability while women care for the family, limiting the possibilities for women to foster a career, nurture their personal interests, and be financially independent. Indeed, the care of an IMD patient occupies many hours a day, forcing the main parent/caregiver—in most cases a woman, as just mentioned—to reduce or stop working. This generates yet another issue: a loss of income and therefore financial difficulties for the family [10].

Many surveys have been performed over the years to assess the medical and social requirements of patients with a rare disease, but no studies have focused specifically on European or IMD patients. While the current literature offers precious information about the general needs of those with a rare disease, which certainly overlaps at least in part with the needs of IMD patients, more specific and detailed information is needed about the social and psychological requirements of patients with a rare metabolic disease living in Europe.

To support and disseminate good healthcare practices in the field of IMDs, in 2017 the European Reference Network for Hereditary Metabolic Disorders (MetabERN) was established. MetabERN includes 78 centres specialised in rare metabolic diseases from 23 EU Member States; the network also involves 44 patient organisations and is endorsed by the Society for the Inborn Errors of Metabolism (SSIEM). Overall, MetabERN cares for almost 33,000 IMD patients. In an attempt to obtain a comprehensive overview of the social and psychological status and needs of IMD patients, especially in Europe, MetabERN has developed and distributed a dedicated survey among metabolic patients.

## Results

### Study population

A total of 924 people responded to the survey: about 1/3 of them were patients (31%), while the majority were parents of patients with a rare disease (60.3%), and only a small portion were caregivers (8.7%). Most of the respondents were females (64.2%) (Table 1). Survey participants were from 25 European countries, with Spain, Italy, and Germany being the most represented; only eight participants were outside of Europe (Honduras, Argentina, Guatemala, Mexico, Taiwan, Australia) (Table 1). The age of the patients was quite evenly distributed between children (under 18 years of age; 51.4%) and adults (over 18 years old; 48.6%) (Table 1). The most represented metabolic diseases affecting the patients were amino and organic acids-related disorders (AOA; 35.4%) and lysosomal storage disorders (LSD; 28.7%) (Table 1).

**Table 1:** Characteristics of the survey respondents

Question and answer options	%
Type of respondent	
Parent of the patient	60.3
Patient	31.0
Caregiver/relative of the patient	8.7
Sex of respondent	
Female	64.3
Male	35.7
Age of patient	
Under 18	51.4
Over 18	48.6
Rare metabolic disorder affecting the patient	
AOA	35.4
LSD	28.7
C-FAO	10.9
Not metabolic	8.1
CDG	5.0
NOMS	1.7
PM-MD	1.6
PD	0
No answer	5.4
Undefined	1.6
Uncertain	1.5
Country of the respondent	
Spain	17.7
Italy	17.2
Germany	15.4
France	9.8
Norway	8.3
Denmark	5.0
Slovakia	4.3
No answer	4.2
Ireland	3.0
Netherlands	2.6
Greece	2.1
Poland	1.9
United Kingdom	1.7
Belgium	1.5
Portugal	1.0
Sweden	1.0
Austria	0.9
Other Europe	1.4
Extra Europe	0.9

AOA: Amino and organic acids-related disorders; PM-MD: disorder of pyruvate metabolism, Krebs cycle defects, mitochondrial oxidative phosphorylation disorders, disorders of thiamine transport and metabolism; C-FAO: carbohydrate, fatty acid oxidation and ketone bodies disorders; LSD: lysosomal storage disorders; PD: peroxisomal disorders; CDG: congenital disorders of glycosylation and disorders of intracellular trafficking; NOMS: disorders of neuromodulators and other small molecules

### Schooling and intellectual disability

The fact that about half of the patients were children was somehow reflected in the fraction of patients currently in school: 48.5% in school versus 51.5% not in school (Table 2). The majority of those in school attended public institutions (65.5%), with a small fraction attending public schools specifically designed for children with assistance needs (16%) or private schools (9.4%) (Table 2).

**Table 2:** Availability of social services

Question and answer options	%
Is the patient in school?	
Yes	48.5
No	51.5
What type of school does the patient attend?	
Public school	65.5
Public school specifically designed for children with assistant needs	16.0
Private school	9.4
Other	9.1
Does the patient suffer from intellectual disability?	
Yes	28.9
No	71.1
Are the services of a social worker guaranteed?	
Yes	27.8
No	38.2
I do not know	34.0
Has the patient obtained civil disability?	
Yes	62.8
No	37.2
On the board that determines civil disability, is the presence of an expert doctor in rare diseases required?	
Yes	29.8
No	32.7
Do not know	37.5
In case of a disabled person in a family, is there any economic support provided to the disabled person by the local, regional or national government, such as:	
Discount on bills, taxes, etc.?	
Yes	25.5
No	51.2
I do not know	23.6
Allowance for independent living?	
Yes	20.0
No	39.0
I do not know	41.0
Allowance for non-self-sufficiency?	
Yes	33.9
No	30.0
I do not know	36.1
Are job opportunities made available in the public sector for patients with various types of limitations?	
Yes	36.2
No	11.9
Do not know	52.0
In the workplace, is part-time/flexible schedule possible (e.g. home-based work)?	
Yes	16.0
No	16.5
Do not know	67.5
Have central or local governmental institutions developed “After Us” projects?	
Yes	5.8
No	24.2
Do not know	70.0

**Fig. 1:**
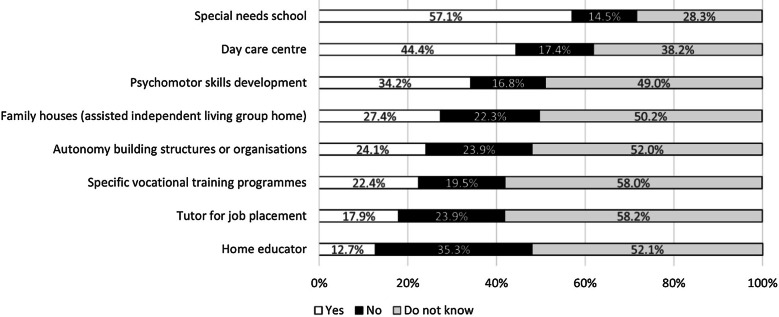
Free educational and development programs offered in the respondent’s country by any government department or non-government institution in the case of mental disability

Most of the survey participants (71.1%) reported no intellectual disability affecting the patient (Table 2). For those with a mental disability, when asked about the kind of free educational and development programs offered in their country by a public or private institution, the majority of respondents (57.1%) said they are offered a special needs school (Fig. 1). Other types of services, such as a home educator, day care centre, psychomotor skills development, autonomy building structures, tutor for job placement, vocational training programmes, and family houses are available for a limited number of patients (from a minimum of 13% to a maximum of 44%, Fig. 1); importantly, about half of the respondents do not know about these services (Fig. 1). At a national level, most of the aforementioned educational and development services are available for the majority of patients in the Netherlands and Belgium, while most patients in Italy and Slovakia report no availability or no knowledge of such services (Additional file 1, Figure S1).

### Social worker services

Regarding the availability of social workers’ services, most respondents replied that such services are not guaranteed (38.2%) or they have no knowledge on the matter (34%), with only about 28% of respondents saying that social workers’ services are guaranteed (Table 2). The situation varies a lot from country to country: the majority of respondents from France (86%) said there are no social worker services, most of those from Slovakia (74%) have no knowledge about such services, while most patients in Denmark (79%) report social services availability (Additional file 1, Figure S1).

### Civil disability and economic support

Most patients (62.8%) have obtained recognition of civil disability (Table 2). When asked if the presence of a rare disease expert was required in the board that decided on such disability, the majority replied “No” (32.7%) or “I do not know” (37.5%) (Table 2). Regarding the economic support provided to the disabled person by the local, regional, or national government, only a small portion of respondents (between 20 and 34%) said that there is availability of discounts on bills or other expenses and/or allowances for independent living or non-self-sufficiency (Table 2). Importantly, a significant portion of participants (between 25 and 41%) do not know about these types of economic help (Table 2).

### Job opportunities

**Fig. 2:**
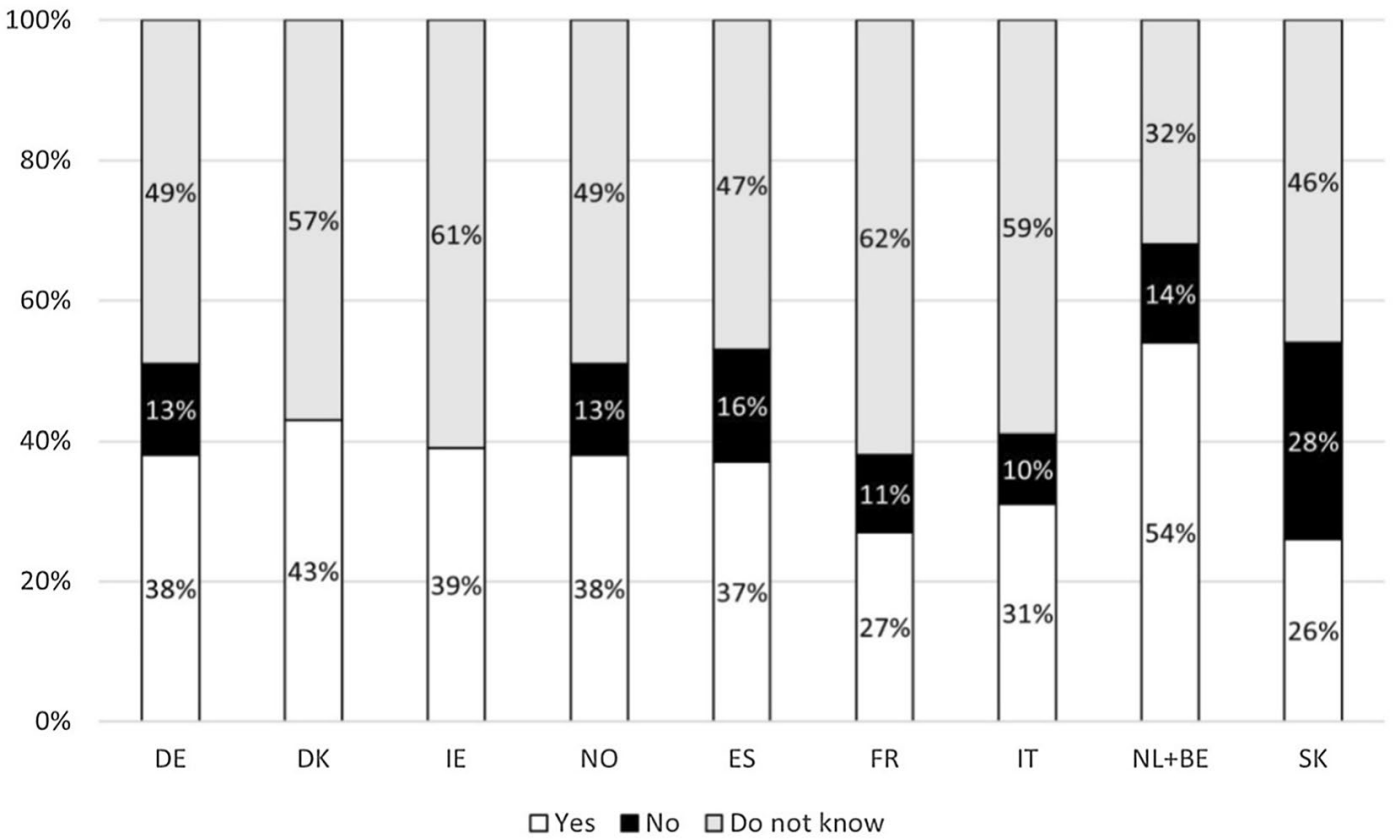
Availability of job opportunities in the public sector for patients with various types of limitation in selected countries (%). DE: Germany; DK; Denmark; ES: Spain; FR: France; IE: Ireland; IT: Italy; NL + BE: Netherlands + Belgium; NO: Norway; SK: Slovakia

Job opportunities in the public sector for people with various types of limitations were available for 36.2% of patients; over half of the respondents (52%) replied that they do not know about these possibilities (Table 2). These results were quite homogenous across the European countries that were took into consideration, with the Netherlands and Belgium being the only exception: here almost 54% of respondents reported public job opportunities for disabled people (Fig. 2).

In the workplace context, only 16% of survey participants reported the availability of part-time and/or flexible schedules (e.g., home-based work); a similar percentage (16.5%) said that these schedules are not possible, while 65.7% of respondents do not know (Table 2).

### Transition from childhood to adult care

**Fig. 3:**
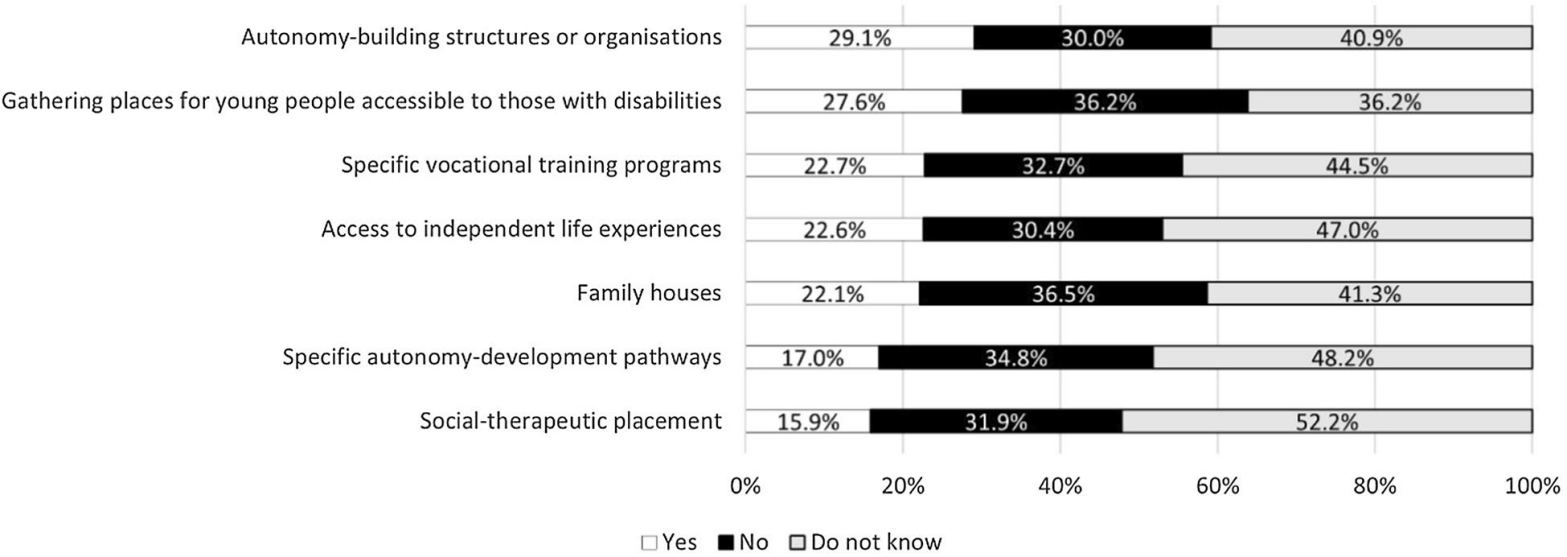
Services and spaces accessible to people with a rare disease regarding the transition from childhood to adulthood

Our respondents reported limited services to support the transition from childhood to adulthood, as these were available for only 16–29% of cases: most participants were unaware of or were not offered gathering places for young people easily accessible by those with disabilities, autonomy-building structures, autonomy-development pathways, vocational training programmes, social therapeutic placement, family houses, or independent life experiences (Fig. 3).

### Psychological support and “after us” projects

**Fig. 4:**
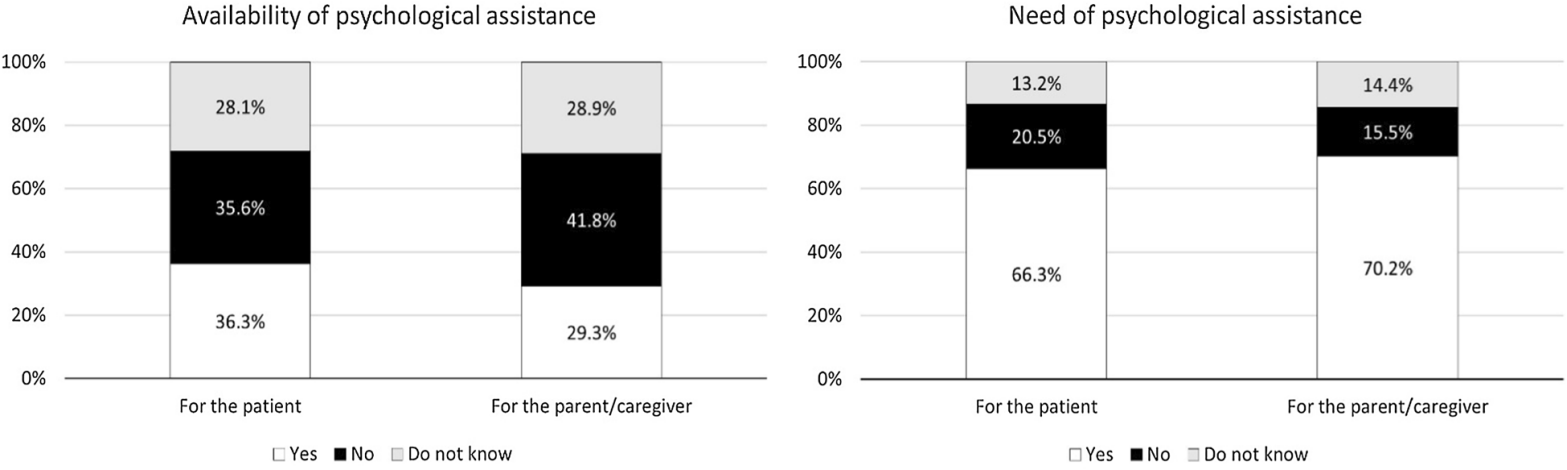
Availability and need of psychological support for the patients and the parents/caregivers (%)

A limited fraction of the respondents confirmed the availability of psychological assistance for the patient (36.3%) or the parent/caregiver (29.3%), despite the fact that the majority considered this type of support necessary both for the patient (66.3%) and the caregiver (70.2%) (Fig. 4).

“After us” projects are specifically designed to address the issue of care for the patient after his/her parents, relatives, or caregivers have died or are incapacitated. When asked if governmental institutions have developed this type of projects, the vast majority of respondents (70%) replied “I do not know” and only about 6% answered “Yes” (Table 2), with no relevant differences from country to country (Additional file 1, Figure S2).

### Specialised medical assistance

In regard to medical support, most of the respondents reported a lack of specialised, disorder-specific medical services (61.1%) or emergency services specialised in their disease (72.3%) in their area (Table 3). However, the majority of respondents (66.5%) said that there is a dedicated clinical pathway for their disorder (from diagnosis to treatment) in their country, and almost 70% said that their metabolic consultant/specialist or a member of their team is easily accessible (Table 3).

**Table 3:** Availability of medical services

Question and answer options	%
Are there disorder-specific medical services, specialised in your disorder, available in your area?	
Yes	34.9
No	61.1
I do not know	4.0
Are there emergency services, specialised in your disorder, in your area?	
Yes	19.0
No	72.3
I do not know	8.7
In your country's health system, is there a particular pathway dedicated to your disorder area, in which a patient is assisted from diagnosis up to starting treatment, to follow-ups and annual tests, etc.?	
Yes	66.5
No	23.0
I do not know	10.5
Is your metabolic consultant/specialist or a member of their team easily accessible?	
Yes	69.5
No	30.5
Is home care necessary for the patient?	
Yes	24.7
No	75.3
What is your level of satisfaction with medical home examinations/appointments?	
Satisfied	31.6
Neither satisfied nor dissatisfied	36.2
Dissatisfied	32.2
What is your level of satisfaction with scheduled home visits?	
Satisfied	27.7
Neither satisfied nor dissatisfied	37.8
Dissatisfied	34.5

A quarter of the study participants (24.7%) reported the need of home care (Table 3). Of these, only about 1/3 is satisfied with the medical home examinations/appointments (31.6%) or scheduled home visits (27.7%).

### Medical expenses

In most cases, medical costs for medical devices, medications, food supplements/dietary integrators, physiotherapy, and speech therapy are partially or fully covered by the national health system (Table 4). However, between 20 and 28% of respondents said that they do not know if/how medical devices, physiotherapy or speech therapy are covered by their healthcare system (Table 4). In addition, respectively 36% and 45% of participants do not know about psychological or home care assistance coverage (Table 4). Also, no coverage was reported for food supplements/dietary integrators or psychological assistance in 30% and 21% of cases, respectively (Table 4).

**Table 4:** Coverage of medical expenses by national healthcare (HC) systems

	Fully covered by HC system (%)	Partially covered by HC system (%)	Not covered by HC system (%)	Costs covered by patient/ no profit ass. (%)	Do not know (%)
Medical devices (e.g., crutches, wheelchair, adapted bed, etc.)	33.1	33.5	8.1	1.0	24.2
Medications	50.4	43.7	3.1	0.5	2.3
Food supplements/dietary integrators	26.2	27.9	29.2	0.7	16.1
Physiotherapy	36.2	29.6	13.1	1.2	19.9
Speech therapy	36.9	19.7	14.7	0.4	28.3
Home care assistance	17.4	22.3	14.6	0.9	44.8
Psychological assistance	25.1	15.6	20.9	2.5	35.9

Looking at the medical costs data from the national perspective, Slovakia emerged as the country where most of our respondents were unaware of costs coverage by the healthcare system, with over half saying that they do not know if/how the health system covers physiotherapy, speech therapy, home care or psychological assistance, and between 42 and 47% admitting to not knowing if medical devices or food supplements/dietary integrators are covered (Additional file 1, Table S1). At the same time, in Spain almost 1/4 of respondents reported no coverage by the healthcare system for medical devices, and about 1/3 (30–37%) said there is no coverage for food supplements/integrators, physiotherapy, speech therapy or psychological assistance (Supplementary material). Based on the data provided by our study participants, Germany, Denmark, and Ireland emerged as the countries where the majority of medical expenses are fully covered by the health system (Additional file 1, Table S1).

**Fig. 5:**
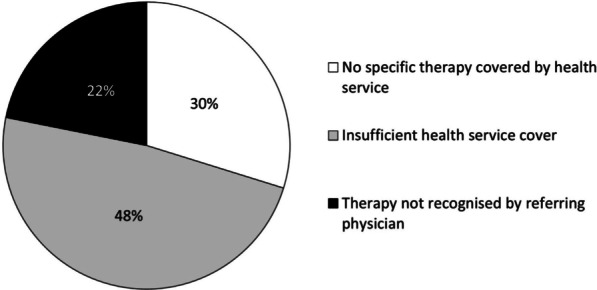
Reasons for choosing private sector’s services

Our survey also investigated the reasons that drive IMD patients or their parents/caregivers to use services from the private sector. The main reasons identified were: insufficient health service coverage (48.3%), no specific therapy covered by the health system (29.8%) and need of therapy not recognised by the referring physician (21.9%) (Fig. 5).

## Discussion

This was the very first survey designed to collect information about the social and medical needs of IMD patients, with a particular focus on Europe. With over 900 participants from more than 20 countries, this study provides the most updated and comprehensive overview of the current status of metabolic patients and their families/caregivers from a social, medical, and economic perspective.

Almost all study participants were from 25 European countries, with Spain, Italy, and Germany being the most represented. As such, our survey collected replies from IMD patients cared by very different national health and social systems, making our results sufficiently diversified to provide a reliable picture of the average European situation. This allows us to provide a general view that takes into consideration local differences in care. However, our data represents only the patients’ and caregivers´ point of view and it is possible that some of the services covered by our survey are actually available locally or nationally, but the patients/caregivers are not aware of them.

In our survey the majority of respondents were females. This confirms other studies which showed a major involvement of women as primary carers and also confirms the general higher involvement of women in health and on-line surveys [11]. This is a reflection of an old-fashioned patriarchal view of society in which women are expected to take care of the family. Since patients with a rare disease require many hours of care every day, creating a heavy physical and psychological burden on the parent or caregiver, in the current situation many women are forced to quit their job and put aside their career prospects, often neglecting their own healthcare and developing depressive symptoms and feelings of isolation [11, 13]. Clearly, more needs to be done to provide daily assistance to rare disease and IMD patients in order to alleviate the burden on caregivers and, consequently, to allow women to fulfil their personal and professional goals and gain a better position in society.

In this study, AOA and LSD were the most represented metabolic diseases. This is probably due to a higher dissemination of the survey among AOA and LSD patients by their patient organisations, and is not to be considered as a reflection of a higher incidence of those diseases.

The age of the patients involved in our survey was quite evenly distributed between children and adults, providing a good overview of the medical and social services offered to IMD patients during their whole lifetime, not only during a specific phase. In addition, the age distribution of our respondents represents an indirect confirmation of the fact that more and more IMD patients are reaching the adult age thanks to better management and/or treatment of their disease, which was not possible until recently [14].

According to our results, the majority of metabolic patients attend normal public schools, with only 16% of patients attending a public institution specifically designed for children with assistance needs. This might represent a positive aspect that suggests the possibility for IMD patients to interact with other children and reach a good level of integration with their peers; at the same time, such situation may be the result of a widespread lack of local schools for children with special needs. Children with an IMD often develop physical and/or mental disability, so their education requires special assistance, from appropriate transport or desks, to learning support to help them develop new skills. Not all public schools are able to provide these types of services, and when they do the quality is variable.

For IMD patients with mental disability, special needs schools are available for over half of the patients, but other free educational services are not widely accessible: home educators, day care centres, psychomotor skills development, autonomy building structures, tutors for job placement, vocational training programmes, and family houses are available for a limited number of patients, with the majority of respondents saying that they do not know about this type of services. Local and national governments need to greatly improve the educational services for patients with intellectual disability, as they are key to support their development and allow them to achieve their full potential and become independent adults. These services are also a great help for families and caregivers, as they alleviate the burden of continuous care. In this respect, the European Commission should also promote some policy changes and/or funding programmes to ensure that rare disease patients receive all the educational services they need in the Member States. Where free educational services are already in place, more effort is needed to disseminate the information among the rare disease community and make sure that these services reach IMD patients.

Only 28% of respondents said that social workers services are guaranteed, with the majority reporting no guaranteed service or no knowledge on the matter, which indicates that currently the majority of IMD patients do not receive any assistance from social workers. Like other rare disease, IMDs have a severe impact on everyday life, from affecting the ability to perform daily tasks and personal care, to limiting motor and sensorial functioning; as such, the burden of IMDs is heavy for both the patients and their carers [11]. In this context, the widespread lack of support from social services represents an important gap in the care of rare metabolic patients that needs to be solved at a local or national level. Particular attention is required also on the training of social service workers: according to a recent survey performed on the wider rare disease community, most patients consider social services professionals poorly prepared to support them [11]. Therefore, appropriate training of care professionals should be part of the effort to improve the lives of IMD patients.

Civil disability has been recognised for most of the patients involved in the survey. Only a small fraction said that the presence of a rare disease expert in the committee that decided on the disability was required; however, about 37% of the respondents said that they do not know about this aspect, so the real figures might be higher. In any case, rare metabolic diseases are complex disorders that are not well known by the general medical community and require expertise to fully understand their implication on the health and life of the patient and his/her caregivers. Therefore, IMD experts should always be included in the disability committee that evaluates a patient with a rare metabolic disease.

Many EU Member States have introduced quotas to encourage the hiring of disabled people in the private and public sector [15]. However, over half of the respondents report no knowledge of reserved job opportunities in the public sector for patients with various types of limitations, so more needs to be done to disseminate this information among the rare metabolic community. According to a previous survey involving all types of rare diseases, over 70% of patients feel insufficiently informed about their social rights or the help they could be entitled to [11]; our results are in line with these findings. In this regards, patients’ organisations could be involved to collect and disseminate the relevant information at a local and/or national level.

Sixteen percent of respondents said that they have the possibility of flexible work, and about the same percentage said that this is not possible. The majority of respondents (~ 66%) report no knowledge on the matter. About half of the patients involved in the survey are children, so these data might reflect the fact that most patients and their parents have not reached the working age yet, so they have no experience nor information on the topic. However, considering that about half of the patients involved in the survey are adults, the percentage of those that have access to flexible work is still limited. IMDs are complex and their manifestations can vary widely and unexpectedly over a short period of time, so flexible working arrangements are essential to allow an adult with a metabolic disease to be independent and manage a career while taking care of his/her disease. In this respect, political actions together with social awareness campaigns are needed. Compared to a recent survey on rare disease patients which reported 35% of respondents with part-time jobs [11], our data on work flexibility is worse. Since that study included patients with any rare disease, while our study focused only on those with IMDs, our results may be due to the low availability of job opportunities locally, where the respondents live.

Activities, programmes, and places to allow or accompany the transition of metabolic patients from childhood to adulthood are very limited and scarce. As highlighted by another recent publication [16], there is a clear need to raise awareness on the importance of this process and to provide specific programmes and services to help rare disease patients become independent and productive adults.

Among the general rare disease community, a major need is associated to services that help patients to maintain their autonomy and self-management of the disease, including psychological care (47%), support to adapt a house to their needs (30%), medical devices (28%) and adapted transports (23%) [11]. Our survey was more specific, as it looked at what services are available for IMD patients with intellectual disability and for the transition from paediatric to adult age; in both cases, our results show a lack of services to support the autonomy of IMD patients. Regarding the availability of psychological support, our data highlight a critical lack of psychological assistance for both the patients and their parents/caregivers, despite the high need of such support. This is not surprising, as IMDs require constant monitoring and care, with numerous visits to different specialists and adjustments of diet and treatment over time; this, together with the disadvantages of an eventual disability and physical pain, often results in stress and depression [12]. In addition, being affected by a rare disease that is largely unknown creates a sense of isolation which worsens depression [12, 13]. Indeed, rare diseases are reported as having a huge impact on mental health, with 37% of patients saying that they feel unhappy or depressed often or very often, and 34% saying that in the past month they were not able to overcome their problems [11]. Urgent actions are required to provide psychological support to patients and parents/caregivers in the rare disease community, including IMDs, as this emerged as a major unmet critical need.

“After us” projects address the issue of care for the patient after his/her parents or caregivers have died or have become incapacitated. Our survey shows that these projects are widely unknown among the IMD community. Dedicated actions are required to raise awareness on the topic at a political and social level; at the same time, the information already available on “After us” projects should be disseminated more effectively. To this end, a collaboration between local healthcare, social services and patient organisations would surely help.

According to our study, for most IMD patients no general nor emergency disorder-specific services are available locally, but national clinical pathways are defined, and medical professionals of reference are readily available when needed. Despite the need for implementation in national organisation and local services for people affected by IMDs, our data show that the necessary basic healthcare and support is generally guaranteed. The large and rapid accessibility of specialists or someone from their team highlights the existence of a trustful and reliable relationship between patients and their families and the metabolic experts, which suggest the existence of a strong IMD community that does its best to support the patients despite the lack of local infrastructures/services. This confirms other findings from a recent study about the impact of COVID-19 on IMD patients [16], which also emphasised the presence of a solid IMD community that acted swiftly during the first phase of the coronavirus emergency to support and protect the patients.

For those who need home care, only a small portion of respondents is satisfied with the home medical examinations and appointments. Even if there is a formal availability of health professionals for home care, our findings suggest that the actual service provided to patients is insufficient and needs major improvements, both in terms of frequency and quality.

Among the general rare disease community, most patients consider the costs associated with their illness to be high and difficult to manage, with around 50% saying that economic benefits or reimbursements were the least met needs [11]. We can confirm those findings only partially based on the data collected on the costs coverage by local healthcare systems. Indeed, according to our results national health services in Europe cover all or part of the expenses for medications, medical devices, food supplements, dietary integrators, physiotherapy, and speech therapy for rare metabolic patients, although significant gaps still exist: between 1/3 and 1/4 of respondents reported no coverage or no awareness of costs coverage for medical devices, food supplements/integrators and speech therapy, and this fraction was even higher for psychological and home care assistance. This highlights a clear gap in communication between the patients and healthcare providers/system, who should offer complete information about the metabolic disease, not only from a clinical, but also from an administrative and economic point of view. In this respect, patient associations can be extremely helpful in collecting and sharing information. In a context where access to appropriate treatment and assistance is essential to guarantee the wellbeing of metabolic patients and their caregivers, financial barriers can have deleterious effects. Despite obvious differences from country to country, improvements need to be made across all Europe to fill current healthcare gaps and ensure that a complete, holist care is provided to all metabolic patients.

Considering the complexity and chronicity of metabolic disorders, covering the basic medications and therapies to avoid the physical deterioration of the patient is not enough. Psychological and home care assistance are also important and require major improvement by all national health and social services. This applies not only to IMD patients, but also to their parents or caregivers. Additional support from local or national services is required to help patients to achieve independence and to reduce the load currently carried by the parents or caregivers; this extra support is essential to allow both IMD patients and their caregivers to be more present at work and at social events, thus allowing them to live healthier, more productive, and inclusive lives.

## Conclusions

Although this survey was conducted on a relatively small population of patients and caregivers, it is the first study focused on metabolic patients. Our data show a widespread lack of social, psychological, and economic support for IMD patients in Europe, which is in accordance with other studies conducted on patients affected by different rare diseases. Local and national governments need to greatly improve their services for rare metabolic patients, as these are important to allow them to achieve their full potential and become independent adults. This assistance is also a great help for families and caregivers, as it alleviates the burden of continuous care. In this respect, political actions together with social awareness campaigns are needed. Since it is possible that some of the services covered by the survey are actually available locally or nationally, but the patients are not aware of them, more effort is needed to disseminate this information among the rare metabolic community. To this end, patients’ organisations should be involved to collect and disseminate the relevant information at a local and/or national level. We believe that this study might also to be considered valuable for the identification of points of actions to be implemented in the EU National Plans for Rare Diseases.

## Methods

The survey was translated in 12 languages and shared with the community of rare metabolic disease patients using the websites and social media of MetabERN and its members and partners (e.g., healthcare providers and patient associations). The survey was designed using the Survey Monkey platform to collect the 924 anonymous completed questionnaires. The survey covered 46 questions with the scope of collecting information on the perception of social and medical assistance in the daily life of rare metabolic patients (Additional file 2) and for this reason it was addressed to adult patients or caregivers (i.e. patients over the age of 18 and caregivers of patients under 18 years of age or with intellectual disability). The survey was active for nine weeks, from 13th April to 15th June 2020. Data was extracted using SPSS, while descriptive and more advanced statistics analysis were performed with Microsoft Excel and SAS. For analysis at the national level, only the data of those countries for which replies from at least 20 participants were collected were taken into consideration.

## Supplementary Information

**Additional file 1**.** Table S1**. National breakdown of costs coverage by healthcare systems (%) per main countries of responders.** Figure S1**. Social services for IMD patients and free educational/development programs for people with mental disability in selected countries.** Figure S2**. “After us” projects developed by governmental institutions. A) Overall responses; B) Responses per selectedc countries. DE: Germany; DK; Denmark; ES: Spain; FR: France; IE: Ireland; IT: Italy; NL+BE: Netherlands+Belgium; NO: Norway; SK: Slovakia.

**Additional file 2**. Perception of Social Assistance in the Daily Life of Rare Metabolic Patients.

## Data Availability

The datasets used and/or analysed during the current study are available from the corresponding author on reasonable request.

## Abbreviations

IMD: Inherited metabolic diseases
MetabERN: European reference network for rare hereditary metabolic disorders
