# Supplementary material for: Social and medical needs of rare metabolic patients: results from a MetabERN survey
Source: Orphanet J Rare Dis. 2021 Aug 3;16:336. doi: 10.1186/s13023-021-01948-5 (PMC8329639; doi:10.1186/s13023-021-01948-5)

**Supplementary Material**

Table S1: National breakdown of costs coverage by healthcare systems (%) per main countries of responders.

|  | **DE** | **DK** | **IE** | **NO** | **ES** | **FR** | **IT** | **NL+BE** | **SK** |
| --- | --- | --- | --- | --- | --- | --- | --- | --- | --- |
| **Medical devices (e.g., crutches, wheelchair, adapted bed, etc.)** | | | | | | | | | |
| Fully covered by HC system | 54.1 | 56.2 | 76.9 | 75.6 | 5.6 | 13.9 | 34.4 | 33.3 | 7.7 |
| Partially covered by HC system | 31.1 | 31.3 | 7.7 | 12.2 | 41.1 | 60.5 | 12.9 | 52.4 | 46.2 |
| Not covered by HC system | 2.7 | 6.3 | 7.7 | 0.0 | 24.4 | 2.3 | 7.5 | 0.0 | 0.0 |
| Costs covered by patient/no profit association | 0.0 | 0.0 | 0.0 | 0.0 | 1.1 | 2.3 | 0.0 | 0.0 | 3.8 |
| Do not know | 12.2 | 6.3 | 7.7 | 12.2 | 27.8 | 20.9 | 45.2 | 14.3 | 42.3 |
| **Medications** | | | | | | | | | |
| Fully covered by HC system | 67.0 | 57.1 | 70.6 | 54.2 | 13.9 | 68.3 | 57.4 | 34.6 | 54.3 |
| Partially covered by HC system | 31.1 | 32.1 | 23.5 | 40.7 | 80.0 | 30.0 | 38.0 | 61.5 | 25.7 |
| Not covered by HC system | 1.9 | 10.7 | 0.0 | 0.0 | 1.7 | 1.7 | 1.5 | 3.8 | 11.4 |
| Costs covered by patient/no profit association | 0.0 | 0.0 | 5.9 | 0.0 | 0.9 | 0.0 | 0.0 | 0.0 | 0.0 |
| Do not know | 0.0 | 0.0 | 0.0 | 5.1 | 3.5 | 0.0 | 3.1 | 0.0 | 8.6 |
| **Food supplements/dietary integrators** | | | | | | | | | |
| Fully covered by HC system | 20.9 | 21.4 | 46.2 | 26.8 | 16.7 | 38.3 | 38.0 | 23.8 | 3.3 |
| Partially covered by HC system | 30.8 | 32.1 | 15.4 | 41.1 | 22.6 | 25.0 | 19.0 | 57.1 | 26.7 |
| Not covered by HC system | 39.6 | 32.1 | 30.8 | 17.9 | 37.2 | 31.7 | 21.5 | 14.3 | 23.3 |
| Costs covered by patient/no profit association | 0.0 | 0.0 | 7.7 | 0.0 | 1.0 | 0.0 | 0.0 | 0.0 | 0.0 |
| Do not know | 8.8 | 14.3 | 0.0 | 14.3 | 22.6 | 5.0 | 21.5 | 4.8 | 46.7 |
| **Physiotherapy** | | | | | | | | | |
| Fully covered by HC system | 59.0 | 70.6 | 40.0 | 23.1 | 13.4 | 53.8 | 38.6 | 33.3 | 6.9 |
| Partially covered by HC system | 21.7 | 23.5 | 33.3 | 53.8 | 27.8 | 21.2 | 21.8 | 45.8 | 31.0 |
| Not covered by HC system | 7.2 | 5.9 | 6.7 | 7.7 | 30.9 | 5.8 | 13.9 | 0.0 | 6.9 |
| Costs covered by patient/no profit association | 0.0 | 0.0 | 13.3 | 0.0 | 3.1 | 0.0 | 0.0 | 0.0 | 0.0 |
| Do not know | 12.0 | 0.0 | 6.7 | 15.4 | 24.7 | 19.2 | 25.7 | 20.8 | 55.2 |
| **Speech therapy** | | | | | | | | | |
| Fully covered by HC system | 65.1 | 68.8 | 40.0 | 40.6 | 11.2 | 52.9 | 38.2 | 47.1 | 7.1 |
| Partially covered by HC system | 12.7 | 12.5 | 40.0 | 31.3 | 22.5 | 15.7 | 9.8 | 41.2 | 17.9 |
| Not covered by HC system | 4.8 | 12.5 | 0.0 | 3.1 | 30.3 | 9.8 | 12.7 | 5.9 | 10.7 |
| Costs covered by patient/no profit association | 0.0 | 0.0 | 10.0 | 0.0 | 1.1 | 0.0 | 0.0 | 0.0 | 0.0 |
| Do not know | 17.5 | 6.3 | 10.0 | 25.0 | 34.8 | 21.6 | 39.2 | 5.9 | 64.3 |
| **Home care assistance** | | | | | | | | | |
| Fully covered by HC system | 39.6 | 41.7 | 18.2 | 34.4 | 3.6 | 2.4 | 14.3 | 57.1 | 0.0 |
| Partially covered by HC system | 35.8 | 33.3 | 27.3 | 21.9 | 23.8 | 16.7 | 14.3 | 33.3 | 19.2 |
| Not covered by HC system | 1.9 | 0.0 | 0.0 | 6.2 | 21.4 | 21.4 | 19.4 | 0.0 | 3.8 |
| Costs covered by patient/no profit association | 0.0 | 0.0 | 18.2 | 0.0 | 1.2 | 0.0 | 1.0 | 0.0 | 0.0 |
| Do not know | 22.6 | 25.0 | 36.4 | 37.5 | 50.0 | 59.5 | 51.0 | 9.5 | 76.9 |
| **Psychological assistance** | | | | | | | | | |
| Fully covered by HC system | 46.2 | 15.8 | 16.7 | 35.7 | 17.6 | 23.7 | 24.8 | 47.8 | 7.1 |
| Partially covered by HC system | 10.8 | 21.1 | 25.0 | 26.2 | 15.7 | 16.9 | 7.1 | 39.1 | 10.7 |
| Not covered by HC system | 4.6 | 31.6 | 16.7 | 7.1 | 33.3 | 27.1 | 24.8 | 4.3 | 7.1 |
| Costs covered by patient/no profit association | 0.0 | 5.3 | 16.7 | 0.0 | 4.6 | 0.0 | 2.7 | 0.0 | 0.0 |
| Do not know | 38.5 | 26.3 | 25.0 | 31.0 | 28.7 | 32.2 | 40.7 | 8.7 | 75.0 |

DE: Germany; DK; Denmark; ES: Spain; FR: France; IE: Ireland; IT: Italy; NL+BE: Netherlands+Belgium; NO: Norway; SK: Slovakia.

Figure S1: Social services for IMD patients and free educational/development programs for people with mental disability in selected countries.


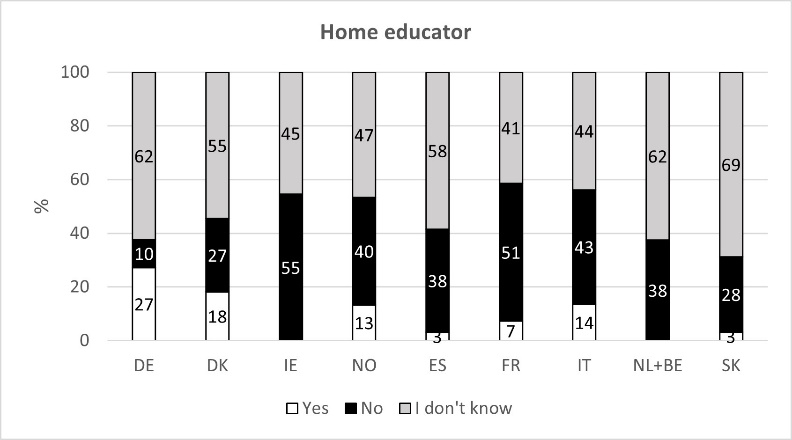

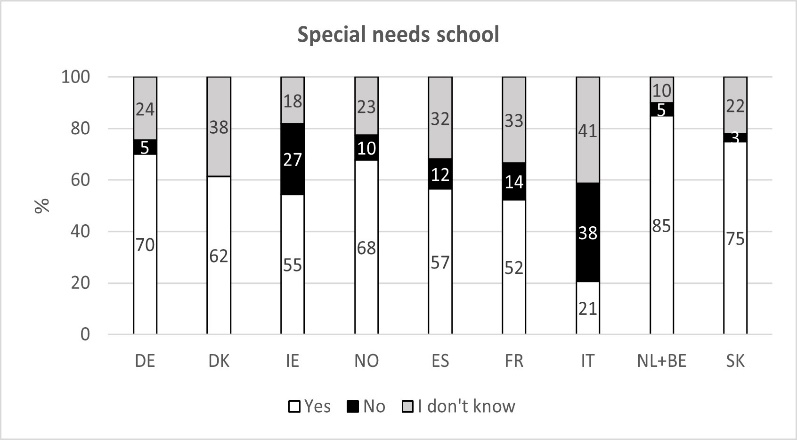

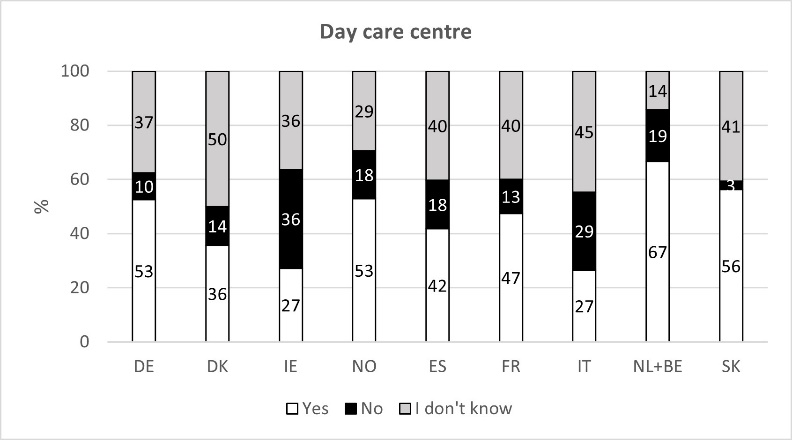

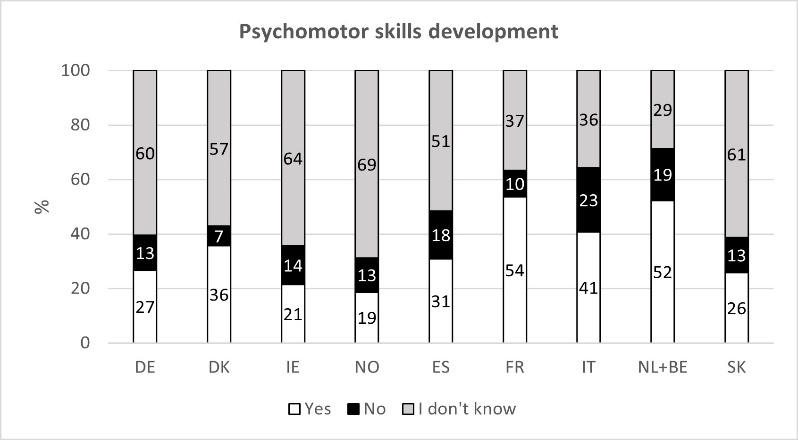

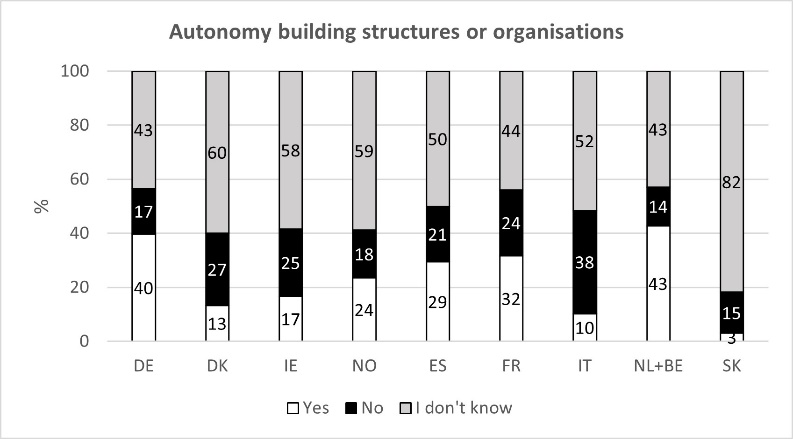

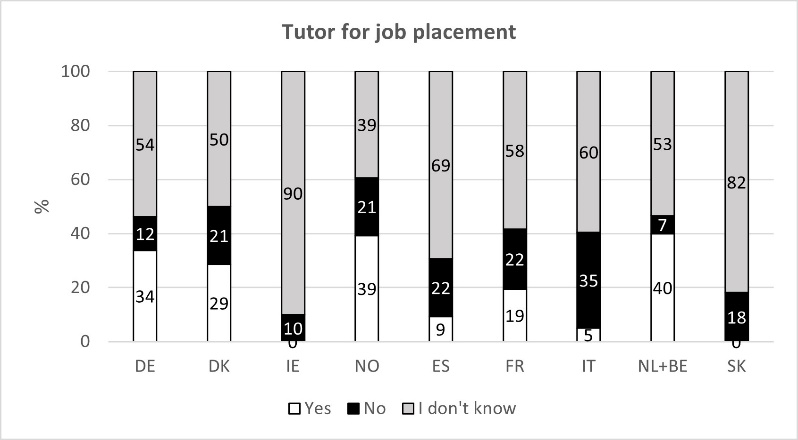

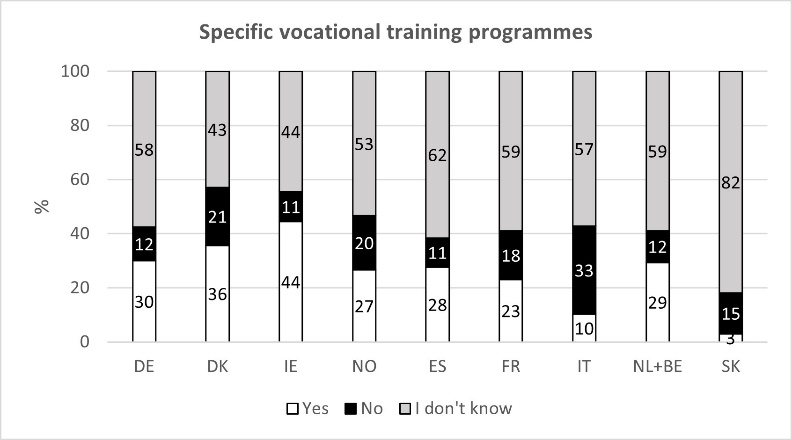

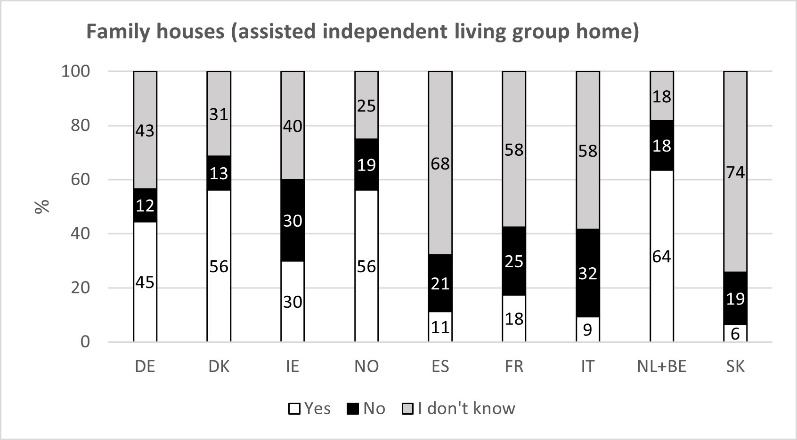


Figure S2: “After us” projects developed by governmental institutions. A) Overall responses; B) Responses per selected countries. DE: Germany; DK; Denmark; ES: Spain; FR: France; IE: Ireland; IT: Italy; NL+BE: Netherlands+Belgium; NO: Norway; SK: Slovakia.


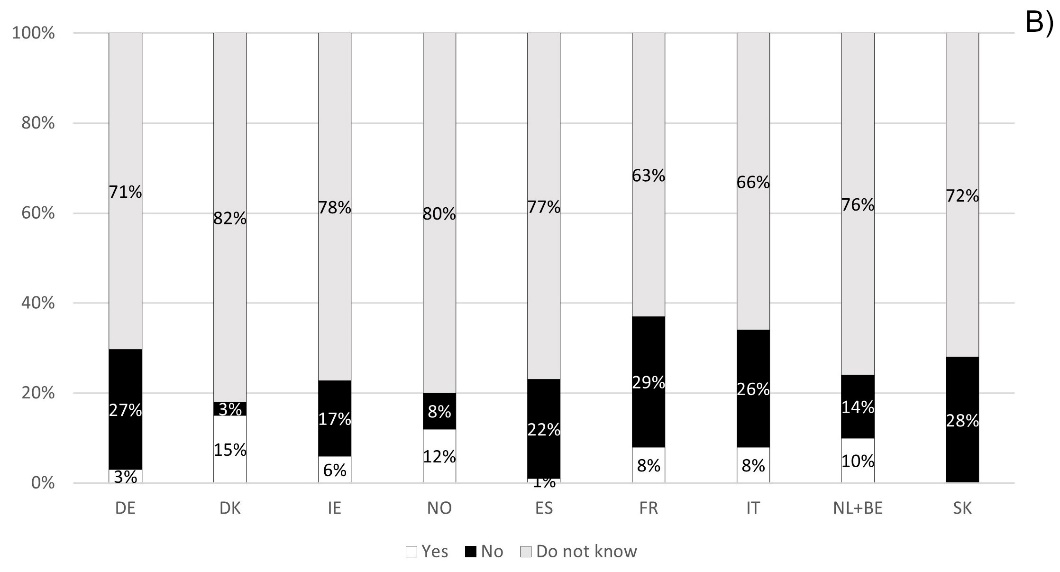

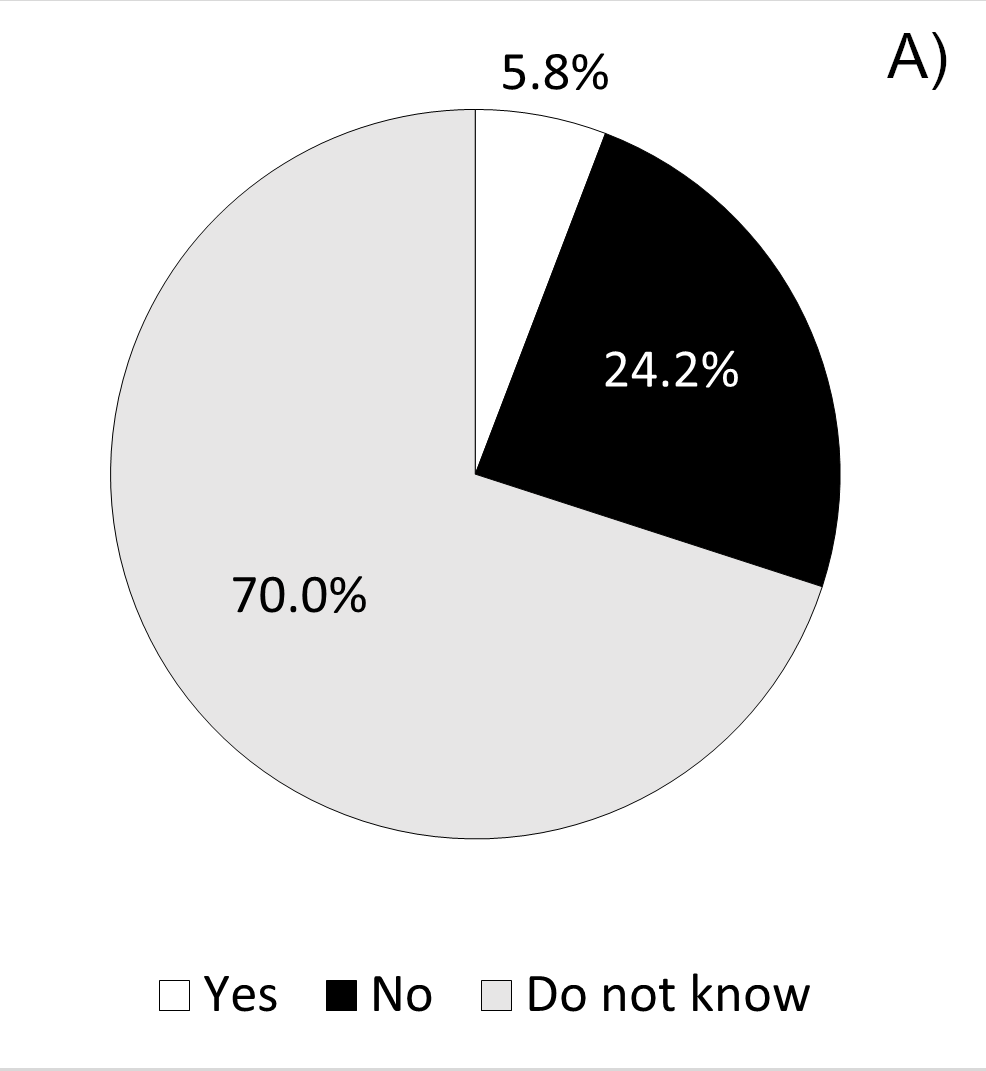

Supplement: Supplementary file 1 — Additional file 1. Table S1. National breakdown of costs coverage by healthcare systems (%) per main countries of responders. Figure S1. Social services for IMD patients and free educational/development programs for people with mental disability in selected countries. Figure S2. “After us” projects developed by governmental institutions. A) Overall responses; B) Responses per selectedc countries. DE: Germany; DK; Denmark; ES: Spain; FR: France; IE: Ireland; IT: Italy; NL+BE: Netherlands+Belgium; NO: Norway; SK: Slovakia. [file 13023_2021_1948_MOESM1_ESM.docx]
