# Supplementary material for: Social and medical needs of rare metabolic patients: results from a MetabERN survey
Source: Orphanet J Rare Dis. 2021 Aug 3;16:336. doi: 10.1186/s13023-021-01948-5 (PMC8329639; doi:10.1186/s13023-021-01948-5)
Supplement: Supplementary file 2 — Additional file 2. Perception of Social Assistance in the Daily Life of Rare Metabolic Patients. [file 13023_2021_1948_MOESM2_ESM.pdf]

Perception of Social Assistance in the Daily Life of Rare Metabolic Patients

Introduction

Welcome to the survey on patients' perspectives on social assistance needs.

"Quality of life is very important for patients, and in particular for patients who suffer from rare or unknown disorders"

Who is this survey for?

All rare metabolic patients and/or their caregivers can participate, as long as they are at least 18 years old. Only one survey per patient.

What is the main aim of this survey?

To understand the differences in social assistance needs of patients affected by rare metabolic disorders in Europe.

What will the main outcomes of this study be?

- **to have a clear picture of the European social assistance landscape/measures regarding rare metabolic patients;**
- **to know the social assistance needs of patients;**
- **to inform patient associations on this topic and empower them to lobby for social assistance measures in their country.**

How long will it take me to complete this survey?

The survey contains 46 questions and should take roughly 15 minutes to complete.

By completing the survey to the best of your ability, you will help us to build a complete and representative data set for these important purposes.

Who developed this study?

This survey was developed by MetabERN, the European Reference Network on Rare Hereditary Metabolic Disorders and particularly by the members of Work Package 6.

Learn more about MetabERN HERE.

What will you do with my data?

All answers are anonymous. Answers will be collected and analysed by the MetabERN central office. The individual responses will not be shared with any third-party entities, with the exception of Survey Monkey. At the end of the analysis, the conclusions will be returned to the healthcare providers and patient associations in MetabERN, and if the study is conclusive then it may be published and/or a report will be posted on the MetabERN website. The findings of the study will be shared with other ERN teams and the European Commission officers (DG Santé). The Survey Monkey privacy policy and cookie policy is accessible at the bottom of the survey home page.

Do you have any further questions?

Contact cinzia.bellettato@metab.ern-net.eu

Deadline:

By clicking "agree" you confirm:

- **you have read and understood the previous information;**
- **you voluntarily agree to participate;**
- **you are at least 18 years of age;**
- **you are a rare metabolic patient yourself or a family member/caregiver of someone living with a rare metabolic disorder.**

* 1. Please indicate your choice below:

☐ Agree

☐ Disagree, and by doing so I refuse to participate in the survey

Perception of Social Assistance in the Daily Life of Rare Metabolic Patients

* 2. Are you?

◯ A patient

◯ A parent

◯ A caregiver/relative of a patient

* 3. Are you?

- Male
- Female

* 4. What country do you live in?

* 5. What is the name of the rare metabolic disorder that you/or the patient is affected by?

* 6. How old are you (the patient)?

- Under 18
- Over 18

Perception of Social Assistance in the Daily Life of Rare Metabolic Patients

Your town/city

This section is to gather information about services offered in the place that you live.

7. In your city/town, is there a reference contact point (office, institution) for access to local, regional or national government benefits and services?

- Yes
- No
- I don't know

8. Are public buildings accessible to disabled people?

- Yes, all
- Some
- No
- I don't know

9. Are there disorder-specific medical services, specialised in your disorder, available in your area?

- Yes
- No
- I don't know

10. Are there emergency services, specialised in your disorder, in your area?

- ◯ Yes
- ◯ No
- ◯ I don't know

11. In your country's health system is there a particular pathway dedicated to your disorder area, to which a patient is assisted from diagnosis up to starting treatment, to follow-ups and annual tests, etc?

- ◯ Yes
- ◯ No
- ◯ I don't know

12. Is your metabolic consultant/specialist or a member of their team easily accessible?

- ◯ Yes
- ◯ No

13. Do you (the patient) need food supplements or dietary supplements?

- ◯ Yes
- ◯ No
- ◯ I don't know

14. In your (the patient) case, is home care necessary?

- ◯ Yes
- ◯ No

Perception of Social Assistance in the Daily Life of Rare Metabolic Patients

15. Is the cost of home care covered by the government?

- ◯ Fully
- ◯ Partially
- ◯ Not at all

Perception of Social Assistance in the Daily Life of Rare Metabolic Patients

16. What is your level of satisfaction with the following aspects of home care?

	Very dissatisfied	Dissatisfied	Neither satisfied nor dissatisfied	Satisfied	Very satisfied
Range of care offered	○	○	○	○	○
Having blood and other types of samples collected (e.g. urine) at home	○	○	○	○	○
Medical home examinations/appointments	○	○	○	○	○
Scheduled home visits	○	○	○	○	○
Physiotherapy	○	○	○	○	○
Other types of at home therapy	○	○	○	○	○

Other types of at home therapy (please specify)

Perception of Social Assistance in the Daily Life of Rare Metabolic Patients

17. Are the services of a social worker guaranteed?

- ○ Yes
- ○ No
- ○ I don't know

Perception of Social Assistance in the Daily Life of Rare Metabolic Patients

18. Rate your satisfaction with **social work services**.

Very dissatisfied	Dissatisfied	Neither satisfied nor dissatisfied	Satisfied	Very Satisfied
○	○	○	○	○

Perception of Social Assistance in the Daily Life of Rare Metabolic Patients

19. Is psychological assistance available?

	Yes	No	I don't know
For the patient	◯	◯	◯
For parents/caregivers	◯	◯	◯

20. Do you think that psychological assistance is necessary?

	Yes	No	I don't know	Not applicable
The patient	◯	◯	◯	◯
Parents/caregivers	◯	◯	◯	◯

21. Are physiotherapy services easily accessible in specific medical facilities, such as hospitals, physiotherapy centres, private institutions?

- ◯ Yes
- ◯ No
- ◯ I don't know
- ◯ Not applicable

22. If you use a physiotherapy service, how many sessions a week do you do?

- ◯ Less than 1
- ◯ 1
- ◯ 2-3
- ◯ 4-5
- ◯ More than 5
- ◯ Don't use

Perception of Social Assistance in the Daily Life of Rare Metabolic Patients

23. If you do not use the physiotherapy service, how many sessions per week would you need?

- ◯ Less than 1
- ◯ 1
- ◯ 2-3
- ◯ 4-5
- ◯ More than 5
- ◯ Don't know

24. Are all necessary physio-therapies (e.g. pre or post-rehabilitation) approved and made available by the health service or must they be carried out on a privately-paid basis?

- ◯ Fully covered
- ◯ Partially covered
- ◯ Not covered
- ◯ Don't know
- ◯ Not applicable

25. In the case of using the private sector, why is it necessary to do so?

	Yes	No	Don't know	Not applicable
There is no specific therapy covered by health service	◯	◯	◯	◯
Health-service cover is insufficient	◯	◯	◯	◯
The need for that therapy is not recognised by the referring physician	◯	◯	◯	◯

26. Do you (the patient) suffer from an intellectual disability?

- ◯ Yes
- ◯ No

27. In the case of mental disability, what kind of educational and development programs are offered in your country, at no cost, by any government department or non-government institution/organisation?

	Yes	No	I don't know	Not applicable
A home educator	○	○	○	○
Special needs school	○	○	○	○
A day care centre	○	○	○	○
Psychomotor skills development	○	○	○	○
Autonomy building structures or organisations	○	○	○	○
Tutor for job placement	○	○	○	○
Specific vocational training programmes	○	○	○	○
Family houses (assisted independent living group home)	○	○	○	○

Other, any service provided by any government you perceive as appropriate to the education/development of the patient (please specify)

[ ]

Perception of Social Assistance in the Daily Life of Rare Metabolic Patients

School-age patients

This section is for school-age patients.

28. Is the patient at school?

- ○ Yes
- ○ No

Perception of Social Assistance in the Daily Life of Rare Metabolic Patients

29. What type of school does the patient attend?

- ◯ Public school
- ◯ Public school specifically designed for children with assistance needs
- ◯ Private school (Fee paying)
- ◯ Other (please specify)

30. In the case of a private school, is the choice due to

- ◯ Reasons unrelated to the disease
- ◯ Greater facilities related to the disease, compared to public school
- ◯ There is no suitable public school in your region
- ◯ Not applicable
- ◯ Other (please specify)

31. We are interested in your views on the following elements of the school facilities which the patient attends

	Not applicable	Very dissatisfied	Dissatisfied	Neither satisfied nor dissatisfied	Satisfied	Very satisfied
The transportation services to and from the school are properly equipped	◯	◯	◯	◯	◯	◯
Does the school canteen provide for the dietary needs of the patient	◯	◯	◯	◯	◯	◯
How happy are you with the quality of the teaching the patient receives.	◯	◯	◯	◯	◯	◯
If you are aware of an anti-bullying policy at the school are you satisfied with its implementation.	◯	◯	◯	◯	◯	◯

32. Is extra assistance related to the patient’s condition provided, for the following?

	Yes	No	I don't know	Not applicable
pre-school feeding assistance	◯	◯	◯	◯
junior school feeding assistance	◯	◯	◯	◯
senior school feeding assistance	◯	◯	◯	◯
transport service for the school	◯	◯	◯	◯

Other (please specify)

Perception of Social Assistance in the Daily Life of Rare Metabolic Patients

Transition from childhood to adulthood, and available facilities and jobs

This section is for patients transitioning from childhood to adulthood as well as those who are adults and are looking for work.

33. Is the patient transitioning from childhood to adulthood?

◯ Yes

◯ No

Perception of Social Assistance in the Daily Life of Rare Metabolic Patients

34. Regarding the transition from childhood to adulthood

	Yes	No	I don't know	Not applicable
Are gathering places for young people in your city accessible to people with disabilities?	◯	◯	◯	◯
Are autonomy-building structures or organisations provided?	◯	◯	◯	◯
Are specific vocational training programs planned/available?	◯	◯	◯	◯
Is social-therapeutic placement offered?	◯	◯	◯	◯
Are family houses available?	◯	◯	◯	◯
Are there specific autonomy-development pathways?	◯	◯	◯	◯
Is there the possibility of access to independent life experiences?	◯	◯	◯	◯
Is there personal psychological support for you (the patient)?	◯	◯	◯	◯
Is there personal psychological support for parents/caregivers/other?	◯	◯	◯	◯

35. Is there any economic support (e.g. special deductions) provided by the local, regional or national government in the case of a disabled person in a family, such as?

	Yes	No	I don't know
discount on electricity bills, heating expenses, taxes etc	◯	◯	◯
allowance for independent living	◯	◯	◯
allowance for non-self-sufficiency	◯	◯	◯

Other (please specify)

[ ]

36. Have you (the patient) obtained recognition of civil disability?

- ◯ Yes
- ◯ No

37. On the board that determines such recognition, is the presence of an expert doctor in rare diseases required?

- ◯ Yes
- ◯ No
- ◯ I don't know

38. Is civil disability recognition necessary to easily obtain services/facilitations like?

	Yes	No	I don't now
awarding of a disabled parking permit	◯	◯	◯
access to specific areas of public transport	◯	◯	◯
accompanying allowance (sum provided by government specifically to pay for a personal assistant)	◯	◯	◯
discounts on the purchase of food products (supplements, etc.)	◯	◯	◯
discounts on the purchase of medical devices	◯	◯	◯

Other (please specify)

39. Are you (the patient) currently employed?

- ◯ Yes
- ◯ No
- ◯ Not applicable

40. How long did it take you (the patient) to find a suitable job position (from the moment you started job hunting for the first time)?

- less than 3 months
- less than 6 months
- less than 1 year
- less than 2 years
- over 2 years
- I'm currently unemployed
- Not applicable

41. Are job opportunities made available in the public sector for patients with various types of limitations?

- Yes
- No
- I don't know

42. What support is given in the workplace for you (the patient) to facilitate your employment?

	Yes	No	I don't know
Are there any workplace facilitations (aids, etc.)?			
Is part-time/flexible schedule possible (e.g. home based work)?			
Are health and hospitalisation needs recognised? (e.g. medical appointments)			

Other (please specify)

43. Have central or local governmental institutions developed “After Us” projects? (Being projects to address the issue of care for the patient after parents/relatives/caregivers have died or are incapacitated)?

- Yes
- No
- I don't know

Perception of Social Assistance in the Daily Life of Rare Metabolic Patients

44. Are these After Us projects or equivalent developed in your country?

	Yes	No	I don't know
By state departments	○	○	○
With the collaboration of other institutions	○	○	○
With the support of patients associations	○	○	○

45. How is the “After Us” projects or equivalent financed?

	Yes	No	I don't know
Completely dependent on the family	○	○	○
Partially dependent on the family	○	○	○
Paid for by the government/public funds	○	○	○
Wholly supported (covered/paid) by patient associations	○	○	○

Perception of Social Assistance in the Daily Life of Rare Metabolic Patients

Final question

Please see question below.

46. Please fill in the table below

	Fully covered by the healthcare system	Partly covered by the healthcare system	Not covered. The patient/family support(s) all the costs	The costs are covered by a patient/non profit association	I don’t know	Not applicable
Medical devices (e.g. crutches, wheelchair, adapted bed, etc)	◯	◯	◯	◯	◯	◯
Medications	◯	◯	◯	◯	◯	◯
Food supplements/dietary integrators	◯	◯	◯	◯	◯	◯
Physiotherapy	◯	◯	◯	◯	◯	◯
Occupational therapy	◯	◯	◯	◯	◯	◯
Speech therapy	◯	◯	◯	◯	◯	◯
Music therapy	◯	◯	◯	◯	◯	◯
Home care assistant	◯	◯	◯	◯	◯	◯
Psychological assistance	◯	◯	◯	◯	◯	◯
